# Clinical implications of EGFR‐associated MAPK/ERK pathway in multiple primary lung cancer

**DOI:** 10.1002/ctm2.847

**Published:** 2022-05-11

**Authors:** Naixin Liang, Zhongxing Bing, Yadong Wang, Xinyu Liu, Chao Guo, Lei Cao, Yuan Xu, Yang Song, Chao Gao, Zhenhuan Tian, Pancheng Wu, Jianchao Xue, Bowen Li, Ziqi Jia, Xiaoying Yang, Yijun Wu, Ruoying Yu, Rui Liu, Xiaoxi Chen, Qiuxiang Ou, Hua Bao, Xue Wu, Zhili Cao, Ji Li, Shanqing Li

**Affiliations:** ^1^ Department of Thoracic Surgery Peking Union Medical College Hospital, Chinese Academy of Medical Sciences Beijing China; ^2^ Peking Union Medical College Chinese Academy of Medical Sciences Beijing China; ^3^ Peking Union Medical College Eight‐Year MD Program Chinese Academy of Medical Sciences Beijing China; ^4^ Nanjing Geneseeq Technology Inc. Nanjing China; ^5^ Department of Pathology Peking Union Medical College Hospital, Chinese Academy of Medical Sciences Beijing China

Dear Editor,

With improvements in lung cancer screening methods, there has been a dramatic increase in the detection of multiple pulmonary nodules (PNs). In multiple primary lung cancers (MPLCs), surgery achieved a 5‐year overall survival rate that varies from 27.4% to 95.8%.^1^ Targeted therapy has been applied to MPLC patients with different responses due to driver mutation heterogeneity.^2^ Meanwhile, two clinical trials are testing immune checkpoint inhibitors for MPLCs (NCT04026841; NCT04047186). Here, we performed whole‐exome sequencing (WES) with a total of 115 multifocal tumour tissues from 32 patients with synchronous MPLCs (sMPLCs). Comprehensive genomic analyses revealed major pathways associated with the development of multifocal tumours, which provided some guidance for the treatment strategy of MPLC patients.

The commonly mutated genes in multifocal PNs included Epidermal Growth Factor Receptor (*EGFR*) (31%)*, BRAF* (17%), *KRAS* (15%) and *RBM10* (9%) (Figure S1A). *EGFR* (31%) was the most frequently mutated gene in multifocal tumours while the primary *EGFR* mutation detected was *EGFR* L858R. Multifocal lung nodules exhibited an increasing disruption of genomic stability with the accumulation of somatic copy number alterations (SCNAs), chromosome instability and tumour mutational burden (TMB) during tumour progression (Figure S1C). Atypical adenomatous hyperplasia (AAH) lesions harboured the fewest SCNAs, while adenocarcinoma (ADC) lesions exhibited extensive chromosome gains and losses across all chromosomes (Figure S1D).

Pairwise analyses were performed with the somatic mutations detected in each sample from the same patient (Supplementary Method). The majority of clinically‐diagnosed multifocal primary tumours were shown as genetically‐different tumours (Table S3). Conversely, 10 patients were identified with more than four shared mutations between multifocal lung nodules, indicating that those tumours were genetically‐similar (GS) and likely evolved from the same origin. Representative cases with genetically similar tumours are shown in Figure 1 with the corresponding computed tomography scans. Phylogenetic tree analyses indicated that all GS tumours had close genetic relationships to each other and were of the same clonal origin. GS‐tumours from the same anatomic location were shown on the Left. Both P02 and P14 have multiple nodules that existed in the right upper lobes. Patient 02 had two GS tumours at distinct developmental stages, one of which was AAH, and one was preinvasive adenocarcinoma in situ (AIS), which were unlikely to be metastatic nodules. In patient 14, 15 of 25 mutations in the P14_T01 ADC tumour were also observed in the P14_T02 ADC tumour, which had 91 mutations. GS‐tumours were also found located at different anatomic locations (Figure 1, Right). In patient 08, P08_T02 (left lower, AIS) and P08_T03 (left upper, ADC) tumours from the left lower lobe and left upper lobe, respectively, were identified with 14 overlapping mutations. P08_T02 also had four overlapping mutations with P08_T04 (Right upper, minimally invasive ADC/MIA) and P08_T06 (left lower, ADC), respectively. P08_T03 had five shared mutations with P08_T06 as well. Almost all tumours sampled in patient 32 displayed significantly overlapping mutations, despite their different physical locations and developmental stages. The exception was sample P32_T06, which had 11 mutations in total.

**FIGURE 1:**
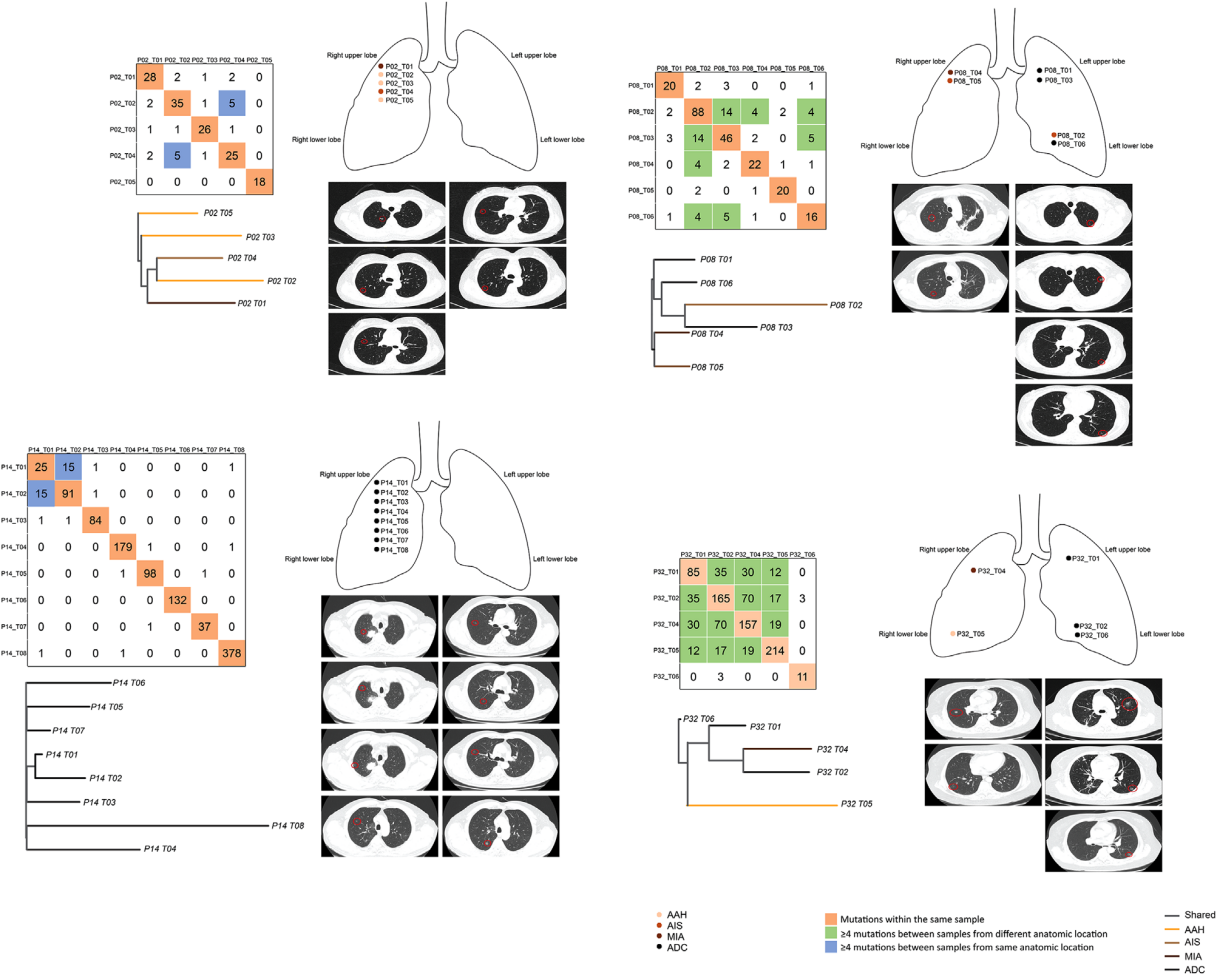
Clinically‐independent but genetically‐similar primary tumours in synchronous multiple primary lung cancers (sMPLCs). Heatmap of mutations shared by lung tumour lesions and the clonal architecture of multifocal tumours. The number of total mutations identified in each tumour (T) from patients (P) with sMPLCs and the number of mutations shared by any pair of lesions were shown. Phylogenetic trees indicated the clonal structure of the sequenced tumour regions in each patient. The developmental stage and tumour location of each lesion were indicated

In this cohort, as shown in Figure 2A, an average of three multifocal lung nodules was sampled from each patient (range: 2–8). Seventy‐two per cent of patients (23/32) had at least one invasive ADC nodule, and nine patients had preinvasive nodules only. Nine patients shared an *EGFR*‐L858R mutation in all nodules. Six cases displayed focal‐specific *EGFR* L858R. Three *EGFR* L858R carriers had a concurrent focal‐specific *EGFR* exon 19deletion (*EGFR* e19del). Two patients had a focal‐specific *EGFR* exon 20 insertion (*EGFR* e20ins). Interestingly, unlike the *EGFR* L858R mutation, the *EGFR* e19del and *EGFR* e20ins driver mutations were focal‐specific only, indicating the association of *EGFR* L858R with multiple primary nodules. Pathway analysis revealed that frequently altered genes during sMPLC development were centered around the Mitogen‐Activated Protein Kinase/Extracellular‐signal‐Regulated Kinase (MAPK/ERK) pathway, including mutated downstream genes and altered upstream activators. In an early stage like AAH, the most enriched alterations were in *MAP2K1* and *BRAF*, while *BRAF* and *EGFR* alterations were the most enriched alterations in the later stages MIA and ADC. This suggested that MAPK/ERK pathway aberrations might be the key factors in the sMPLC tumourigenesis (Figure 2B). We have also employed REACTOME pathway functional enrichment analyses. MAPK associated pathways were among top enriched signaling pathways among different stages of MPLCs (Figure S3). Further studies are warranted to elucidate the role of these mutated MAPK/ERK pathways genes in the tumourigenesis and development of sMPLCs.

**FIGURE 2:**
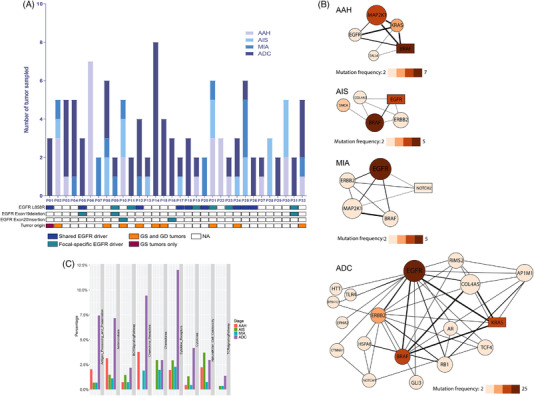
Alterations in *EGFR* and the downstream *MAPK/ERK* pathway were associated with the development of multifocal lung tumours. (A) The developmental stage of the sampled tumours in each patient. *EGFR* driver mutations and the potential for metastasis in each patient are indicated by the bar below the image. Shared EGFR driver was defined as the same EGFR driver identified in more than two nodules of one patient. (B) Pathway analysis of somatic mutations in different developmental stages using the Mutations For Functional Impact on Network Neighbors (MUFFINN) method. (C) Immune‐related pathway analysis of multiple primary lung cancers (MPLCs) at different stages. GS tumours: genetically‐similar. GD tumours: genetically‐different

In this cohort, four patients were treated with chemotherapy, while 26 patients were treated with EGFR tyrosine kinase inhibitors (TKIs) including first‐generation (*n* = 15), second‐generation (n = 2) and third‐generation EGFR‐TKIs (*n* = 9). Two patients received only surgery. Survival analysis suggested that patients with MPLC in EGFR‐TKI therapy might have improved disease‐free survival (DFS) than chemotherapy (Figure S4). However, this result needs further validation due to the limited number of patients with chemotherapy treatment. Meanwhile, we also look into the immune pathway in the MPLCs at different stages. As shown in Figure 2C, immune‐related pathways were more likely to enrich in invasive ADC but not in early‐stage AAH, AIS and MIA, indicating targeting the EGFR‐MAPK pathway might be feasible for MPLC patients. The frequency of *EGFR* mutations in NSCLC is known to differ among different ethnic groups. In a study of 78 synchronous NSCLC patients from Germany*, KRAS* mutations were observed in 25.6% of the cases, a higher rate than that of *EGFR* mutations. In another study of 27 multifocal synchronous ADC patients from US, *KRAS* mutations were identified in 22 cases, while *EGFR* mutations were only identified in two cases.^3^ These molecular features may have some impact on the tumourigenesis and development of MPLCs in different ethnic groups. The result of this study needs to be interpreted with caution when applied to patients of other ethnicities.

Meanwhile, the TMBs of these multifocal tumours were relatively low (median 1.1mut/Mb, Figure S1B), also indicating the sMPLC patients in this cohort were unlikely to respond to immunotherapy. The limited efficacy of PD‐L1 therapy in MPLCs has been observed in different studies.^4^, ^5^ The PD‐L1 therapy may not be successful in the MPLC cohort due to several reasons. First, MPLCs are often diagnosed at early stages and manifested as ground‐glass opacities. The immune microenvironment and escape mechanism of carcinogenesis (AAH to ADC) may be different from that of malignancy development.^6^ Second, MPLC often displayed low TMB, low immune infiltration landscape and low PD‐L1 expression,^5^, ^7^ which may not respond to anti‐PD‐L1 treatment. Although we lack the PD‐L1 information of this enrolled sMPLC cohort, we employed a method (T cell ExTRECT) published in Nature^8^ to estimate the T cell fraction in the WES samples. The median T cell fraction of the sMPLC tumours was 0%, supporting the low T cell infiltration in the tumour samples of sMPLCs (Figure S5). Lastly, *EGFR* mutation in lung cancer has shown an association with a low objective response rate to PD‐L1 inhibitors.^9^ Considering the generally *EGFR*‐mutated, low PD‐L1 expressed, low T‐cell infiltrated profile, EGFR‐TKI as the prior line of therapy may be a better option for the MPLC patient. PD‐L1 inhibitor durvalumab demonstrated efficacy in treating heavily‐pretreated EGFR+ advanced lung cancer patients in the ATLANTIC trial, although the objective response was lower than EGFR‐ group.^10^ Therefore, if the MPLC patient develops resistance to EGFR‐TKIs, PD‐L1 treatment may still be considered at the advanced stage

In conclusion, our data revealed that the development of sMPLC went through MAPK/ERK pathway, which was first described in a large‐scale MPLC sample size. Moreover, patients with EGFR‐TKIs as maintenance therapy achieved a better DFS than patients with maintenance chemotherapy. Combining with the relatively low TMB of this sMPLC cohort, our data indicated that sMPLC patients might benefit from EGFR‐targeted therapy rather than immunotherapy.

## CONFLICT OF INTEREST

Ruoying Yu, Rui Liu, Xiaoxi Chen, Qiuxiang Ou, Hua Bao and Xue Wu are shareholders or employees of Nanjing Geneseeq Technology Inc. The remaining authors have no conflict of interest to declare.

## Supporting information

Supporting Information

Supporting Information

Supporting Information

Supporting Information
