# Supplementary material for: Clinical implications of EGFR‐associated MAPK/ERK pathway in multiple primary lung cancer
Source: Clin Transl Med. 2022 May 11;12(5):e847. doi: 10.1002/ctm2.847 (PMC9091990; doi:10.1002/ctm2.847)
Supplement: Supplementary file 1 — Supporting Information [file CTM2-12-e847-s001.docx]

**Figure S1. Genomic features of multifocal tumors across different developmental stages.** (A) Distribution of arm-level copy number variation and individual gene mutations in the cohort, as assessed by whole exome sequencing. The developmental stage of each lung nodule is provided at the top of the image. Each column represents one patient. (B) Mutational signatures associated with

each stage. The COSMIC mutational signatures were derived from all mutations in each lung nodule. Lung nodules with a minimum

of five unique SNVs were included in the mutational signature deconstruction. (C)Chromosome instability score and tumor mutational burden. Each dot represents the chromosome instability score or tumor mutational burden in each lung nodule. The mean chromosome instability score and tumor mutational burden of all lesions in each histologic stage are shown. (D) Summary plot of chromosome gains and losses in nodules at different histologic stages. Individual chromosomes are shown along the X-axis and the frequency of abnormality is shown along the Y-axis. Red represents chromosome gain and blue represents chromosome loss.

**
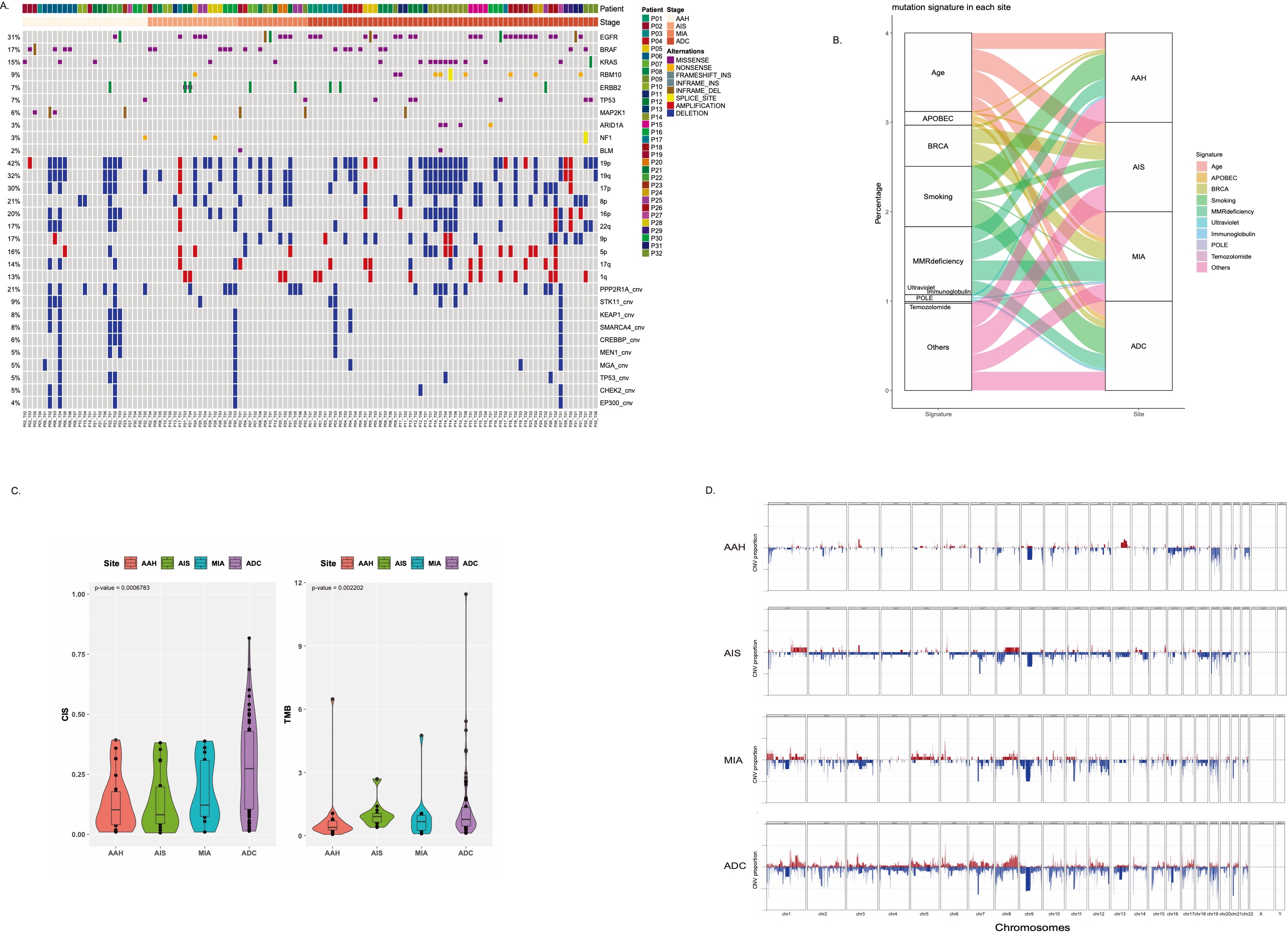
**

**Figure S2. Pairwise comparisons of every two patients were conducted using a published WES data from Chinese lung adenocarcinomas.** Lung nodules with three shared mutations were identified in 0.04% of the total events while lung nodules with two shared mutations in 0.32% and four shared mutations in 0.01% of the total events respectively.

**
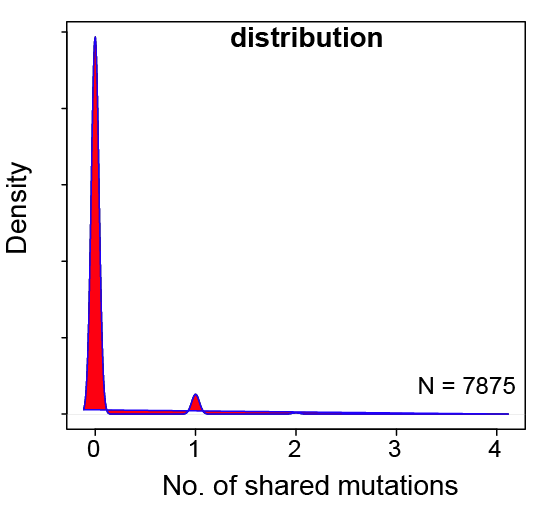
**

| Numbers of shared mutations | 0 | 1 | 2 | 3 | 4 |
| --- | --- | --- | --- | --- | --- |
| Numbers of patients with shared mutations | 7454 | 392 | 25 | 3 | 1 |
| Percentage | 95% | 5% | 0.32% | 0.04% | 0.01% |

**Figure S3. The functional enrichment analyses using REACTOME Pathway databases.** The most frequently mutated 30 genes in each stage were used as input to the REACTOME pathway enrichment analysis R package ReactomePA (version 1.38). Pathways with benjamini-hochberg adjusted p-value smaller than 0.05 were highlighted. For visualization purposes, only the top 20 pathways that were enriched in all four stages samples were displayed.


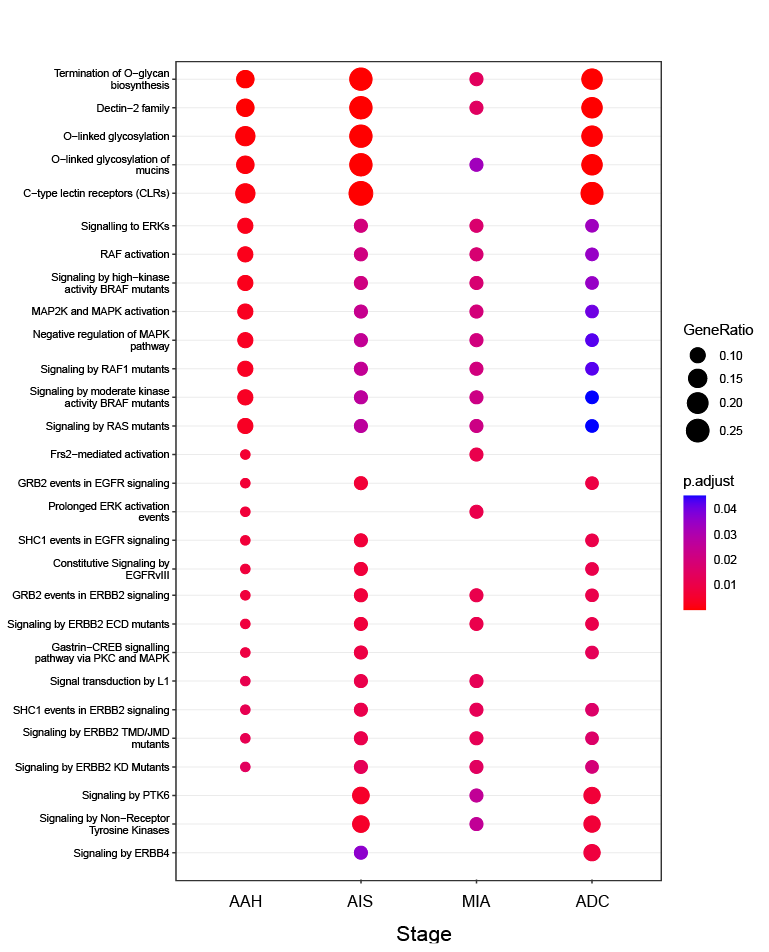


**Figure S4. Kaplan-Meier disease-free survival curves of MPLC patients according to different therapy and GS-tumors.** GS tumors: genetically-similar. GD tumors: genetically-different.


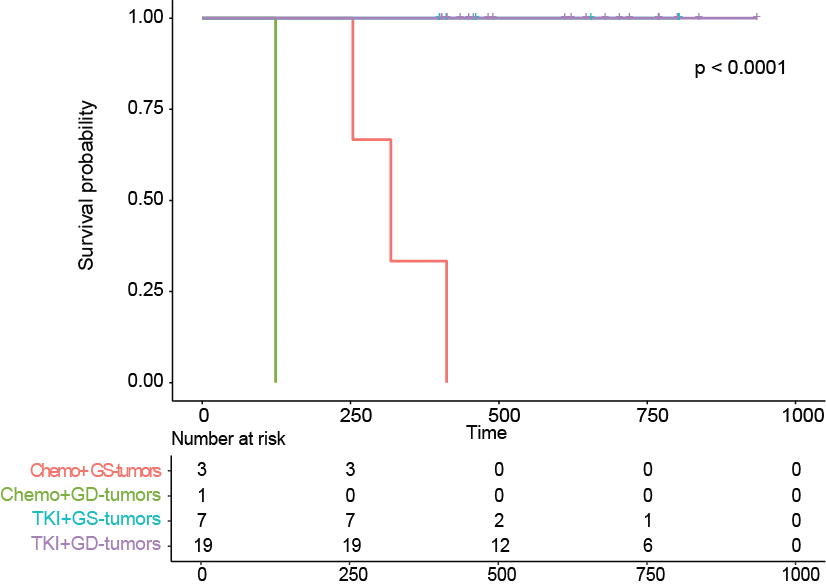


**Figure S5. The T cell fraction in the tumor samples of sMPLC patients.** TCRA: T cell receptor-ɑ gene.


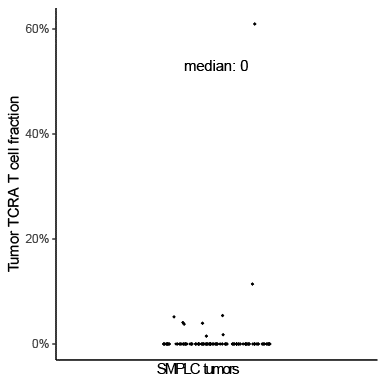


# Table S1. Patient characteristics in the sMPLC cohort

| Characteristics | All patients (N=32) |
| --- | --- |
| Gender - No. (%） | |
| Male | 7 (22%) |
| Female | 25(78%) |
| Age (%) | |
| range | 28-73 |
| median | 56 |
| ≥57 | 18 (57%) |
| <57 | 14 (43%) |
| Smoking History- No. (%) | |
| Smoker | 4(13%) |
| Non-smoker | 28(87%) |
| Maintenance Therapy- No. (%) | |
| EGFR-TKI | 26(81%) |
| Chemotherapy | 4(13%) |
| NA | 2(6%) |
| Characteristics | All samples (N=115) |
| Tumor Stage - No. (%) | |
| AAH | 24 (21%) |
| AIS | 18 (16%) |
| MIA | 14 (12%) |
| ADC | 59(51%) |
| Tumor Site - No. (%) | |
| Left | 35 (30%) |
| Right | 80 (70%) |
| Lobe Location - No. (%) | |
| Upper | 74 (65%) |
| Middle | 6 (5%) |
| Lower | 35 (30%) |
