# Supplementary material for: Clinical implications of EGFR‐associated MAPK/ERK pathway in multiple primary lung cancer
Source: Clin Transl Med. 2022 May 11;12(5):e847. doi: 10.1002/ctm2.847 (PMC9091990; doi:10.1002/ctm2.847)
Supplement: Supplementary file 2 — Supporting Information [file CTM2-12-e847-s004.docx]

**Supplementary Methods**

**Patients and sample information**

Patient information was shown in Table S1 and S2. These patients were clinically diagnosed as synchronized multiple primary lung cancer(sMPLC) according to the Martini and Melamed classification system([Martini and Melamed, 1975](#_ENREF_11)). The American College of Chest Physicians(ACCP) guidelines was also based on the Martini and Melamed classification system([Alberts and American College of Chest, 2007](#_ENREF_1)). The multiple primary lung cancers are defined if 1) tumors physically distinct and separate; 2) tumors of histological different types or tumors of same histology but in different segment, lobe or lung if origin from carcinoma *in situ*, no carcinoma in common lymphatics or no extrapulmonary metastases at the time of diagnosis([Chen et al., 2019](#_ENREF_5)). All patients provided written consent for specimen collection and subsequent genetic testing. A total of 117 resected lung tumor specimens from 32 patients with sMPLC (Table S1) were collected from distinct primary tumors at the Department of Thoracic Surgery, Peking Union Medical College Hospital. WES were performed and two samples were excluded due to QC failure. Among the 32 patients, 78% were female and the median age was 56 years old (range: 28 to 73 years old). The samples included 24 AAH specimens, 18 AIS specimens, 14 MIA specimens, and 59 ADC specimens, of which 30% were from the left lung and 70% were from the right lung. There were 74 samples from the upper lung lobe, 6 samples from the middle lobe and 35 samples from the lower lobe.

**Whole-exome sequencing**

The whole-exome sequencing was performed as previously described([Fang et al., 2019](#_ENREF_9); [Lan et al., 2020](#_ENREF_10)). In brief, genomic DNA from formalin-fixed paraffin-embedded (FFPE) samples and matched normal blood control samples were extracted using the QIAamp FFPE Tissue DNA Extraction Kit and DNeasy Blood and Tissue Kit (Qiagen, USA), respectively, and quantified using a Qubit 3.0 with a dsDNA HS Assay Kit(Thermofisher). DNA libraries were prepared using the KAPA HyperPrep Kit(Roche) and enriched using the xGen Exome Research Panel(Integrated DNA technologies), and Hybridization and Wash Kit. Libraries were then sequenced on an Illumina HiSeq4000 platform using PE150 sequencing chemistry(Illumina). A mean coverage depth of 150X was reached.

**Single nucleotide variant and indel calling**

The sequence data was processed as previously described([Fang et al., 2019](#_ENREF_9)). For quality control procedures, *Trimmomatic* was used to remove low quality samples (quality reading below 20) or N bases from the FASTQ files. Sequencing reads were mapped to the reference human genome (hg19) using the Burrows-Wheeler Aligner. Duplicate reads were removed using *Picard*, followed by realignment around known indels. Base quality recalibration was performed using *GATK4* and samples with Total QScores smaller than 35 or contamination rates greater than 0.02 were excluded. *VCF2LR* was used to remove non-matching samples from matched tumor-normal pairs. *ContEst* was used to estimate cross-sample contamination. Somatic single nucleotide variant (SNV) calling was performed using *Mutect* and insertion/deletions (indels) were called using *Scalpel* (in somatic mode)([Fang et al., 2016](#_ENREF_8)). SNV in the 1000 Genomes Project and dbSNP with frequency >1% were excluded. The SNVs and indels were further filtered as previously described([Fang et al., 2019](#_ENREF_9)).

**Copy number variant(CNV) analysis**

CNV analysis was performed as previously described([Tang et al., 2021](#_ENREF_14)). Tumor purity of the samples were first estimated using ABSOLUTE([Carter et al., 2012](#_ENREF_4)) and then purity-adjusted data were used to calculate gene-level and segment-level CNVs using FACETS([Shen and Seshan, 2016](#_ENREF_13)). Chromosome-level CNV was determined if at least 60% of the given chromosome segments had a consistent level of copy number alterations. For focal CNV events, deep amplifications and deep deletions were counted for further analyses. CNV events were used in calculating the chromosomal instability score, which was defined as the proportion of the length of the genome with segmented copy number alterations.

**Mutational signature analysis**

To define the mutational signatures, we assessed the context of synonymous and nonsynonymous SNVs in 56 resected lung nodules subjected to WES. The normalized mutational patterns were compared to the mutational signatures reported by Alexandrov *et al.*([Alexandrov et al., 2013](#_ENREF_2)). All mutational signatures were confirmed using *deconstructSigs* with default parameters. Only a trend in the increase of APOBEC-associated mutational signatures was identified in different lung tumor types (weigh score: 0.017 in AAH, 0.024 in AIS, 0.057 in MIA, and 0.054 in ADC; Figure S1B) without significant result.

**Statistical analysis**

The Kruskal-Wallis H test was used to compare mutational burdens between tumors, while Jonckheere's trend test was used to compare chromosome instability between tumors. The Cochran–Armitage test was performed to assess the association of arm-level copy number alterations in multifocal tumors. Pathway-centric analyses of somatic mutations from different developmental stages were performed using the Mutations For Functional Impact on Network Neighbors (MUFFINN) method([Cho et al., 2016](#_ENREF_6)). To examine the biological functions of the most frequently mutated genes in each stage, we performed functional enrichment analyses using REACTOME Pathway databases. REACTOME pathway database([Fabregat et al., 2017](#_ENREF_7)) is an integrated database containing advanced functional information for the systematic analysis of gene functions, biological pathways and other research. The most frequently mutated 30 genes in each stage were used as input to the REACTOME pathway enrichment analysis R package ReactomePA([Yu and He, 2016](#_ENREF_15)) (version 1.38). Pathways with benjamini-hochberg adjusted p-value smaller than 0.05 were highlighted. For visualization purposes, only the top 20 pathways that were enriched in all four stages samples were displayed. The Kaplan-Meier survival curves were constructed using R Survminer package and the log-rank test was used to compare survival time between groups. Tumor mutational burden (TMB) was defined as the total number of coding mutations in each sampled lung nodules. The T cell fractions of the tumor samples were calculated using T cell exome TERC tool as previously described([Bentham et al., 2021](#_ENREF_3)), which estimates the T cell fraction from WES samples using a signal from T cell receptor excision circle loss during V(D)J recombination of the T cell receptor-ɑ gene.

To setup a cut-off for molecular characterization of MPLC, pairwise comparisons of every two patients was conducted using a published WES data from Chinese lung adenocarcinomas([Zhang et al., 2019](#_ENREF_16))(Figure S2). Lung nodules with three shared mutations were identified in 0.04% of the total events while lung nodules with two shared mutations in 0.32% and four shared mutations in 0.01% of the total events respectively. Therefore, tumors with less than three mutations were considered as multiple primary tumors of different origin. Whereas tumors with more than four shared mutations (including four mutations) were considered genetically-similar, with the same origin (empirical P < 0.0005). The presence/absence matrix of all mutations and focal CNVs detected in samples were constructed for each patient. The matrixes were then used as inputs for phylogeny reconstruction by the Phangorn package in R with the maximum parsimony algorithm([Schliep, 2011](#_ENREF_12)).

**Reference**

Alberts, W. M., and American College of Chest, P. (2007). Introduction: Diagnosis and management of lung cancer: ACCP evidence-based clinical practice guidelines (2nd Edition). Chest *132*, 20S-22S.

Alexandrov, L. B., Nik-Zainal, S., Wedge, D. C., Campbell, P. J., and Stratton, M. R. (2013). Deciphering signatures of mutational processes operative in human cancer. Cell reports *3*, 246-259.

Bentham, R., Litchfield, K., Watkins, T. B. K., Lim, E. L., Rosenthal, R., Martínez-Ruiz, C., Hiley, C. T., Bakir, M. A., Salgado, R., Moore, D. A.*, et al.* (2021). Using DNA sequencing data to quantify T cell fraction and therapy response. Nature *597*, 555-560.

Carter, S. L., Cibulskis, K., Helman, E., McKenna, A., Shen, H., Zack, T., Laird, P. W., Onofrio, R. C., Winckler, W., Weir, B. A.*, et al.* (2012). Absolute quantification of somatic DNA alterations in human cancer. Nat Biotechnol *30*, 413-421.

Chen, C., Huang, X., Peng, M., Liu, W., Yu, F., and Wang, X. (2019). Multiple primary lung cancer: a rising challenge. Journal of thoracic disease *11*, S523-S536.

Cho, A., Shim, J. E., Kim, E., Supek, F., Lehner, B., and Lee, I. (2016). MUFFINN: cancer gene discovery via network analysis of somatic mutation data. Genome Biol *17*, 129.

Fabregat, A., Sidiropoulos, K., Viteri, G., Forner, O., Marin-Garcia, P., Arnau, V., D’Eustachio, P., Stein, L., and Hermjakob, H. (2017). Reactome pathway analysis: a high-performance in-memory approach. BMC bioinformatics *18*, 1-9.

Fang, H., Bergmann, E. A., Arora, K., Vacic, V., Zody, M. C., Iossifov, I., O'Rawe, J. A., Wu, Y., Jimenez Barron, L. T., Rosenbaum, J.*, et al.* (2016). Indel variant analysis of short-read sequencing data with Scalpel. Nat Protoc *11*, 2529-2548.

Fang, W., Ma, Y., Yin, J. C., Hong, S., Zhou, H., Wang, A., Wang, F., Bao, H., Wu, X., Yang, Y.*, et al.* (2019). Comprehensive Genomic Profiling Identifies Novel Genetic Predictors of Response to Anti-PD-(L)1 Therapies in Non-Small Cell Lung Cancer. Clinical cancer research : an official journal of the American Association for Cancer Research *25*, 5015-5026.

Lan, X., Bao, H., Ge, X., Cao, J., Fan, X., Zhang, Q., Liu, K., Zhang, X., Tan, Z., Zheng, C.*, et al.* (2020). Genomic landscape of metastatic papillary thyroid carcinoma and novel biomarkers for predicting distant metastasis. Cancer science *111*, 2163-2173.

Martini, N., and Melamed, M. R. (1975). Multiple primary lung cancers. The Journal of thoracic and cardiovascular surgery *70*, 606-612.

Schliep, K. P. (2011). phangorn: phylogenetic analysis in R. Bioinformatics *27*, : 592–593.

Shen, R., and Seshan, V. E. (2016). FACETS: allele-specific copy number and clonal heterogeneity analysis tool for high-throughput DNA sequencing. Nucleic acids research *44*, e131.

Tang, W. F., Wu, M., Bao, H., Xu, Y., Lin, J. S., Liang, Y., Zhang, Y., Chu, X. P., Qiu, Z. B., Su, J.*, et al.* (2021). Timing and Origins of Local and Distant Metastases in Lung Cancer. Journal of thoracic oncology : official publication of the International Association for the Study of Lung Cancer *16*, 1136-1148.

Yu, G., and He, Q.-Y. (2016). ReactomePA: an R/Bioconductor package for reactome pathway analysis and visualization. Molecular BioSystems *12*, 477-479.

Zhang, X. C., Wang, J., Shao, G. G., Wang, Q., Qu, X., Wang, B., Moy, C., Fan, Y., Albertyn, Z., Huang, X.*, et al.* (2019). Comprehensive genomic and immunological characterization of Chinese non-small cell lung cancer patients. Nature communications *10*, 1772.
